# Enhancement of Strawberry Shelf Life via a Multisystem Coating Based on *Lippia graveolens* Essential Oil Loaded in Polymeric Nanocapsules

**DOI:** 10.3390/polym16030335

**Published:** 2024-01-26

**Authors:** Barbara Johana González-Moreno, Sergio Arturo Galindo-Rodríguez, Verónica Mayela Rivas-Galindo, Luis Alejandro Pérez-López, Graciela Granados-Guzmán, Rocío Álvarez-Román

**Affiliations:** 1 Departamento de Química Analítica, Facultad de Medicina, Universidad Autónoma de Nuevo León, Monterrey 64460, Nuevo León, Mexico; barbara.gonzalezmrn@uanl.edu.mx (B.J.G.-M.); veronica.rivasgl@uanl.edu.mx (V.M.R.-G.); luis.perezlp@uanl.edu.mx (L.A.P.-L.); graciela.granadosgu@uanl.edu.mx (G.G.-G.); 2 Departamento de Química Analítica, Facultad de Ciencias Biológicas, Universidad Autónoma de Nuevo León, San Nicolás de los Garza 66455, Nuevo León, Mexico; sergio.galindord@uanl.edu.mx

**Keywords:** polymeric coating, essential oil, *Lippia graveolens*, nanoparticles, strawberries

## Abstract

Strawberries (*Fragaria xannanasa*) are susceptible to mechanical, physical, and physiological damage, which increases their incidence of rot during storage. Therefore, a method of protection is necessary in order to minimize quality losses. One way to achieve this is by applying polymer coatings. In this study, multisystem coatings were created based on polymer nanocapsules loaded with *Lippia graveolens* essential oil, and it was found to have excellent optical, mechanical, and water vapor barrier properties compared to the control (coating formed with alginate and with nanoparticles without the essential oil). As for the strawberries coated with the multisystem formed from the polymer nanocapsules loaded with the essential oil of *Lippia graveolens*, these did not present microbial growth and only had a loss of firmness of 17.02% after 10 days of storage compared to their initial value. This study demonstrated that the multisystem coating formed from the polymer nanocapsules loaded with the essential oil of *Lippia graveolens* could be a viable alternative to preserve horticultural products for longer storage periods.

## 1. Introduction

According to the Food and Agriculture Organization (FAO), food losses are defined as a decrease in the quantity or quality of food. An important part of food loss is waste, which is food initially intended for consumption and discarded or, in some cases, used alternatively, i.e., in a non-food way. Globally, between a quarter and a third of the food produced annually for human consumption is lost or wasted. This is equivalent to about 1.3 billion tons of food, including 40 to 50% of roots, fruits, vegetables, and oilseeds [1,2]. The highest losses occur in horticultural products, that is, fruits and vegetables [3]; this is because they are much less resistant and more perishable compared to seeds, tubers, or roots [4]. Strawberries (*Fragaria ananassa* L.) are highly nutritious but, at the same time, are extremely perishable due to their susceptibility to deterioration, mechanical injury, postharvest physiological disorders, and microbial decay [5]. Therefore, strawberries are considered a delicate fruit with a short shelf life: losses during postharvest storage are estimated to be as high as 40% [6]. The use of coatings is an innovative method of food preservation whose application would allow the following: (i) the generation of a physical barrier to protect the surface of the product; (ii) the control of the migration of solutes and humidity as well as, gas exchange and oxidation reactions to prolong shelf life; and (iii) a reduction in the risk of pathogen growth on the surfaces of fruit and vegetable products [7,8].

The principle of using coatings is very similar to modified atmosphere packaging, where an atmosphere consisting of high CO_2_ and low O_2_ concentration is created [9]. This environment can effectively slow down the respiration rate, conserve stored energy, slow microbial growth, and, therefore, extend the shelf life of the fruit [10]. The coating may contain ingredients such as antioxidant agents, additional nutrients, flavorings, preservatives, and antimicrobial compounds of natural origin, such as essential oils (EOs) [11]. Oregano is the common name applied to more than 40 species of the families *Verbenaceae*, *Lamiaceae*, *Compositae*, and *Leguminoseae*, of which the most important are Mediterranean or European oregano (*Origanum vulgare*) and Mexican oregano (*Lippia graveolens*). The antioxidant and antimicrobial properties of *Lippia graveolens* essential oil (EO-*Lg*) [12] make it a strong candidate as a natural preservative for foods, such as fruit and vegetable products. Regarding the chemical mechanism of peroxidation inhibition, the components of the EO act mainly as radical trapping agents that would lead to the formation of hydroperoxides, epoxides, and other oxygenated derivatives. Gutiérrez-Grijalva et al. [13] reported that EO-*Lg* may have significant potential as an auxiliary antioxidant and against enzymes involved in lipid and carbohydrate metabolism. On the other hand, previous studies have shown that EO-*Lg* is effective against bacteria, yeast, and fungi [14] and increased antioxidant activity without negative effects on sensory acceptability in tomatoes [15]. The antimicrobial property of EO-*Lg* is attributed to two of its main components, i.e., thymol and carvacrol [16], which have the ability to break the cell membrane of Gram-negative bacteria. This is thanks to their chemical structure since they contain an -OH group and have a non-polar character. The damage is reflected in the dissipation of the two components of the proton motive force, the pH gradient and the electrical potential, which causes greater permeability and the leakage of ions and other compounds that are necessary for the survival of the bacteria [17]. The antimicrobial effects of oregano EO have been previously reported against microorganisms such as *Candida albicans*, *Alternaria alternate*, *Escherichia coli*, *Pseudomonas aeruginosa*, and *Staphylococcus aureus*, among others [15,18,19]. However, the application of EOs in coatings is often problematic due to their physicochemical properties, and they can deteriorate due to environmental factors, such as light and oxygen, and have high volatility [20]. Furthermore, EOs have low solubility in water, which makes their incorporation into commercial products difficult. To overcome these effects, the nanoencapsulation of EOs in polymeric nanoparticles has become a possible solution [21]. Today, nanotechnology represents an area of opportunity for the development of vehicles that transport certain EOs, vitamins, and other plant extracts, such as polyphenols, with antimicrobial and antioxidant properties. Although the development of nanoparticles (NPs) was first reported in the pharmaceutical field for drug delivery systems less than two decades ago, these systems caught the interest of the food sector. NPs have great potential to guarantee the maintenance of color (by encapsulating dyes), flavor, and nutritional values (by encapsulating vitamins), thus increasing the shelf life of foods [22].

It is possible to produce coatings when antioxidants or antimicrobials are encapsulated in NPs. In the coating, the compound can be administered, prolonged, or controlled to create a specific microenvironment to improve the shelf life of fruit and vegetable products [23]. In addition, a plasticizer such as sodium alginate can be added to the coating; this is widely used because it is biodegradable, biocompatible, has a low price, and is non-toxic. This polysaccharide can form strong and homogeneous films at room temperature [24]. Previous studies have shown that the application of NPs with EOs allowed the color and firmness of the grapes to be preserved and delayed the presence of microbiological damage due to a prolonged storage time [25]. Therefore, the objective of this study was to evaluate the conservative effect of a coating obtained from nanocapsules of *Lippia graveolens* EO (NC-EO-*Lg*) and carry out its optical, mechanical, and permeability characterization for its application in strawberries.

## 2. Materials and Methods

### 2.1. Materials

*Lippia graveolens* was collected from Cuatro Ciénegas, Coahuila de Zaragoza (26°59′ N 102°03′59″ OE), México. The specimens were identified in the Herbarium of the School of Biological Sciences, Universidad Autónoma de Nuevo León, México. Methanol, acetone (Tedia, Fairfield, OH, USA), and isopropyl alcohol (Chromadex, Los Angeles, CA, USA) were of HPLC grade. Purified water was from a Milli-Q water-purification system (Veolia, Boston, MA, USA). The standard solution n-alkanes (C_8_–C_20_, C_22_, and C_24_), myrcene (≥99.5%), p-cymene (≥97%), carvacrol (≥98%), anethole (99%) GC grade, and sodium alginate were purchased from Sigma-Aldrich, St. Louis, MO, USA. Eudragit L100-55 polymer (1:1 methacrylic acid: ethyl acrylate) was purchased from Evonik Industries, Essen, Germany.

### 2.2. Extraction and Physicochemical Characterization of Essential Oil (EO)

#### 2.2.1. Isolation of EO 

For the extraction of EO from *Lippia graveolens* (EO-*Lg*) by hydrodistillation using a modified Clevenger-type apparatus, a quantity of 100 g of the aerial parts of fresh plant was accurately weighed and added to 800 mL of distilled water in a flask. Then, it was placed in a balloon heater attached to a refrigerator to ensure condensation of EO for 4 h. The EO was collected and stored in sealed vials in the dark, at 4 °C, until used. The yield percentage was calculated as the weight (g) of EO per 100 g of the plant [26].

#### 2.2.2. Physical Characterization of EO

The relative density of the EO-*Lg* was determined at 25 °C according to the general method 0251 of the Pharmacopeia of the United Mexican States (FEUM) [27] with an Anton Paar Densimeter (DMA35, Ashland, VA, USA). The refractive index was measured at 25 °C on an Anton Paar Refractometer (Abbermat 300, Ashland, VA, USA) according to Method 0741 of the FEUM [27]. The specific rotation was determined on a polarimeter (Perkin Elmer, Waltham, MA, USA) according to Method 0771 of the FEUM [27]. The relative density and the refractive index test were performed in triplicate, while the specific rotation was performed in sextuplicated. An average value was calculated, and anethole was used as a control.

#### 2.2.3. Chemical Composition of EO Using Gas Chromatography–Mass Spectrometry (GC-MS) and GC with Flame Ionization Detection (GC-FID)

The composition of volatile constituents of the EO-*Lg* was analyzed using a gas chromatograph (Agilent Technologies, 6890N, Santa Clara, CA, USA) equipped with a 5973 INERT mass selective spectrometer (ionization energy 70 eV) and an HP-5MS capillary column (5% phenylmethylpolysiloxane, 30 m × 0.2 mm, 0.25 μm, Agilent J and W, Santa Clara, CA, USA). The ionization-source temperature was 230 °C, the quadrupole temperature was 150 °C, and the injector temperature was 220 °C. Data acquisition was performed in the scan mode. The oven temperature was programmed as follows: 35 °C for 9 min, increased to 150 °C at 3 °C min^−1^ and held for 10 min, increased to 250 °C at 10 °C min^−1^, and increased to 270 °C at 3 °C min^−1^ and held for 10 min. The flow rate of the helium carrier gas (99.999% purity) was 0.5 mL min^−1^. The EO’s components were identified by comparing retention indices relative to C_8_–C_20_, C_22_, and C_24_ n-alkanes, and MS results were compared with the mass spectra from the US National Institute of Standards and Technology (NIST) library and reference data. To determine the proportion of each component, a quantitative analysis was performed with a GC-FID (Autosystem XL, Perkin Elmer, Boston, MA, USA) using the same HP-5MS column. The injector temperature was 270 °C; the oven temperature program was the same as the GC-MS analysis. The percentage composition of each component was calculated using the peak-normalization method.

### 2.3. Preparation and Characterization of the Nanocapsules

The EO-*Lg* nanocapsules (NC-EO-*Lg*) were prepared by the solvent displacement procedure developed by Fessi et al. [28]. The organic phase was prepared by dissolving 175 mg of Eudragit L100-55^®^ and 100 mg of the EO-*Lg* in 15 mL of organic solvent mixture (acetone: isopropyl alcohol 50:50) under magnetic stirring at room temperature. The organic solution was added to 25 mL of the aqueous phase (Milli-Q water) under magnetic stirring (125 rpm). Finally, the organic solvent mixture was then evaporated under reduced pressure (Control Laborota 4003, Heidolph Instruments, Schwabach, Germany). The NP-BCO was prepared by the same method, omitting the EO-*Lg*. Subsequently, the NC physicochemical characterization was determined three times at 25 °C. The mean particle size and polydispersity index (PI) were measured at a 90-degree scattering angle using dynamic light scattering (DLS), while the zeta potential measurement was by laser Doppler microelectrophoresis (Zetasizer Nano-ZS90, Malvern Instruments, Worcestershire, UK). In addition, the size and morphology of the NC were investigated using a scanning electron microscope (SEM), JEOL brand JSM-7401F (Kyoto, Japan), with a field-emission gun (FEG) and an accelerating voltage of 4.0 kV. For this, the NC-EO-*Lg* samples were previously coated with gold-palladium for 15 s using the sputtering technique (Denton vacuum model Desk II equipment, Cherry Hill, NJ, USA). On the other hand, the encapsulation percentage (%E) and the encapsulation efficiency percentage (%EE) of the three main components of the EO-*Lg* (myrcene, p-cymene, and carvacrol) were determined by a previously validated GC-FID method. A calibration curve was made with the mixture of the three standard components of EO-*Lg*, and the retention times were 4.7, 5.4, and 12.2 min, respectively. The detection limits for myrcene, p-cymene, and carvacrol were 0.76, 0.35, and 0.83 µg/mL, respectively. The %E and %EE were calculated by the following formulas:

(1) $$\%E=\frac{\left(mg\;of\;main\;component\;encapsulated\;in\;the\;NC\right)}{\left(mg\;of\;polymer+mg\;of\;main\;component\;in\;total\;EO\right)}\;\times 100$$

(2) $$\%EE=\frac{\left(mg\;of\;main\;component\;encapsulated\;in\;the\;NC\right)}{\left(mg\;of\;main\;component\;in\;total\;EO\right)}\;\times 100$$

### 2.4. Preparation of the Coatings

For the formation of the coatings, the direct casting method [29,30] on Teflon was used, and sodium alginate (AL) powder was incorporated as film-coating into an aqueous suspension of NC-EO-*Lg* under magnetic stirring at 350 rpm. NC-EO-*Lg* coating (NC-EO-*Lg*-AL) was obtained with a sodium alginate concentration of 1% (*w*/*v*) and a drying time of 24 h. The NP-BCO-AL was prepared by the same method, omitting the EO-*Lg*. In addition, the coating’s morphology was investigated using a scanning electron microscope (SEM), JEOL brand JSM-7401F (Kyoto, Japan), with a field-emission gun (FEG) and an accelerating voltage of 2.0 kV. For this, the coating samples were previously coated with gold–palladium for 25 s using the sputtering technique (Denton vacuum model Desk II equipment, Cherry Hill, NJ, USA). 

### 2.5. Physical Characterization of the Coatings

#### 2.5.1. Optical Evaluation

The opacity of the coatings was calculated as the ratio of absorbance (A) (in nm) to the coating thickness (in μm) using a UV–VIS spectrophotometer (Genesys, Thermo Scientific, Waltham, MA, USA). Rectangular coating strips (1 cm × 4 cm) were placed directly in glass cuvettes, using an empty glass cuvette as reference. The absorbance was measured at 600 nm, and the opacity of the coating was calculated by the following equation:

Opacity = A 600 nm/δ(3)

where O = opacity, A 600 nm = absorbance of the sample at 600 nm, and δ = Thickness of the coating in mm. 

#### 2.5.2. Mechanical Evaluation

Mechanical properties such as adhesion, tensile strength, and elongation at break for the coatings were measured by a texturometer (CT3 Texture Analyzer, Brookfield-Ametek, Middleborough, MA, USA). The coatings were cut into strips and gripped at each end with the necessary accessories, and then the jaws were moved at the controlled speed (2 mm/s) until the modulus was automatically recorded.

#### 2.5.3. Fourier-Transform Infrared (FTIR) Spectroscopy Analysis

The chemical composition of the coating (the polymer, NC, and free EO) was analyzed using an FT-IR Optical Frontier Optical Spectrophotometer (PerkinElmer, Waltham, MA, USA). Each coating was scanned 30 times from 4000 to 400 cm^−1^ with a scanning interval of 4 cm^−1^, and the air spectrum was used as a background correction.

#### 2.5.4. Water Vapor Barrier Properties 

The water vapor transmission rate (WVTR) and water vapor permeability (WVP) of the coatings were measured in accordance with ASTM E-96-95 [31]. The determination of the WVTR and WVP were carried out by gravimetric analysis. In a desiccator, coatings were placed on top of a vial containing a saturated solution with a relative humidity (RH) of 95%. Subsequently, the vial was weighed every 2 h for the first 8 h and then at 24 h, in quintuplicate. The water transpiration rate and water vapor permeability were determined with the following formulas [32]:

(4) $$\mathrm{WVTR}=\frac{S}{AT}$$

where S is the weight gain of the test setup (g), A is the area of film exposed, and T is the time (h).

(5) $$\mathrm{WV}P=\frac{\left(WVTR\right)\delta }{Pw(RH1-RH2)}$$

where δ is the average thickness of the films in mm, and Pw is the partial difference of water vapor between the 50% RH of the desiccator and the 0% RH of the saturated solution.

### 2.6. Application of the Coatings on Strawberries

Strawberries with homogeneous characteristics of color, size, and without mechanical damage were selected. They were washed with distilled water and dried. Subsequently, for the coating formation, the spraying method was used, which consisted of spraying the samples of the horticultural product with AL, NP-BCO-AL, or NC-EO-*Lg*-AL for 1 min and then drying them at room temperature [25]. After the application of the coating, the strawberries were stored for 15 days at 4 °C. 

#### 2.6.1. Weight Loss Percentage and Texture Analyses: Penetration Test

The strawberries from the two groups and the control (strawberries without coating) were individually weighed after the storage time at 4 °C. The weight loss was calculated as the difference between the initial and final weights of the fruit, and the values were reported on a percentage basis in accordance with the AOAC standard method. The penetration test was determined by a texturometer (CT3 Texture Analyzer, Brookfield-Ametek, Middleborough, MA, USA). The penetration test outlined a mechanical force–displacement using a 5 kg loading cell and a cylindrical flat head probe with a diameter of 5 mm (P/5) entering the fruit (placed on the plate with the receptacle cavity upright to the compression probe to assess its firmness). The data were acquired with the following instrumental settings: pretest speed, 10.00 mm/s; test speed, 5 mm/s; post-test speed, 10.00 mm/s; trigger force, 2.0 g. For each sample, ten replicates were used.

#### 2.6.2. Visual Microbiological Damage 

On the other hand, the presence of microbiological damage on the strawberries and the control were monitored for the storage time at 4 °C.

### 2.7. Statistical Analysis 

The results obtained in this study are shown in the tables and figures as means ± SDs of different measurements. The statistical differences were evaluated using a one-way analysis of variance (ANOVA) with Tukey’s post hoc test (*p* < 0.05), performed using SPSS software (Version 20.0, SPSS Inc., Chicago, IL, USA).

## 3. Results and Discussion 

### 3.1. Isolation of Essential Oil of Lippia graveolens (EO-Lg)

Initially, the EO-*Lg* was obtained from *Lippia graveolens* by the hydrodistillation method in a Clevenger apparatus. This technique has been commonly used for the extraction of EOs since it avoids the degradation of plant material. The EO is extracted from the plant, together with water vapor, and is separated after condensation. A yield percentage of 5.32% was obtained in this study. Dilworth et al. [33] obtained a similar result, which was 4.29%. The extraction of EOs through hydrodistillation is variable and depends on certain parameters, such as the collection season, soil type, harvest location, and plant variety. On the other hand, lower extraction yields than those obtained in this work have been reported (0.92% to 4.41%) using another extraction technique: steam distillation [34].

### 3.2. Physical Properties of the EO-Lg

For the physical characterization of the EO-*Lg*, the physical parameters of density, refractive index, and optical rotation were evaluated based on the FEUM [27]. In the EO industry, the importance of evaluating the physical characteristics of EOs to guarantee their quality control on a routine basis has been established since variations in physical parameters allow for the possible adulteration or degradation of their components to be detected, therefore ensuring their biological activity [35]. In addition, the anethole standard was selected as a control for physical evaluation due to its physicochemical similarity with some of the components of EO-*Lg* (i.e., polarity and vapor pressure) and the fact that its physical characterization is already reported in the FEUM [27]. In Table 1, the values obtained from the physical characterization of anethole and EO-*Lg* are presented. In the case of anethole, it can be observed that the values obtained are within the intervals reported in the FEUM, which confirms the repeatability and reproducibility of the physical evaluations. 

In the case of EO-*Lg*, the relative density, refractive index, and specific rotation values obtained also coincide with those reported by Torrenegra-Alarcón et al. [36], presenting only small differences, which could be attributed to the geographical origin and the species of the plant. In addition, Domínguez [37] reported that a refractive index greater than 1.47 and a relative density greater than 0.9 indicates that the EO contains oxygenated aliphatic and/or aromatic compounds. These values coincide with what is expected according to Table 2, which shows that more than 99% of the EO-*Lg* compounds are monoterpenes (oxygenated and aliphatic) and aliphatic sesquiterpenes.

### 3.3. Chemical Composition of the EO Using Gas Chromatography–Mass Spectrometry (GC-MS) and GC with Flame Ionization Detection (GC-FID)

**Figure 1:**
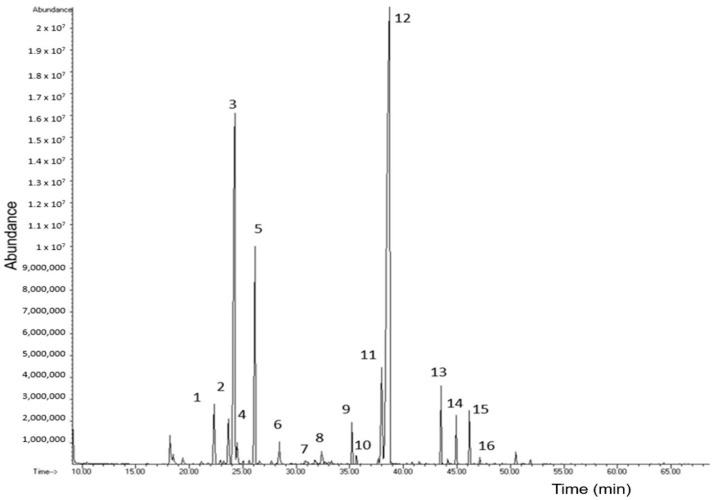
Chromatogram of essential oil of *Lippia graveolens* by gas chromatography–mass spectrometry.

The chemical characterization of EO-*Lg* was carried out using GC-MS and GC-FID. The chromatogram of EO-*Lg* obtained through GC-MS is shown in Figure 1. A total of 16 components were identified in the EO-*Lg* based on a comparison with the NIST (National Institute of Standards and Technology) library, the arithmetic index, and the Kovats index [38]. Next, the percentage abundance (%A) of the 16 components was determined based on their relative areas and is shown in Table 2. 

As is shown in Figure 1, the EO-*Lg* was composed of 24.96% of monoterpenes hydrocarbons and 71.77% of oxygenated terpenes. The major components were myrcene (16.93%), p-cymene (7.56%), and carvacrol (66.58%). This coincides with what was reported by Chacón-Vargas et al. [39], who also identified carvacrol as the majority component, with an abundance percentage of 33.78%. Likewise, in another investigation, carvarol and p-cymene were found to be major components with abundance percentages of 25.19% and 13.78%, respectively [40], which also coincides with what was reported in this study.

However, various authors have reported other major components of EO-Lippia. Such is the case of Capatina et al. [41], who reported thymol (38.82%), p-cymene (20.28%), and γ-terpinene (19.58%) as the majority components. In another study carried out by Sharififard et al. [42], terpineol (22.85%) and α-terpinene (20.60%) were reported as major components. Other authors mention 1,8-cineol as the majority component [43]. The different abundances of the components reported in previous studies are due to variations in the collection season, the year of collection, and the region and/or climate in which the plant grows, as well as the location of the species analyzed and the methods of obtaining the EO such as steam distillation, extraction with organic solvents, ultrasound-assisted extraction, microwave-assisted extraction, and supercritical fluid extraction [44].

In order to monitor the components of EO-*Lg* in the NCs and coating, myrcene, p-cymene, and carvacrol were selected as the monitoring components based on their abundance and antibacterial activity. 

### 3.4. Preparation and Characterization of the NC-EO-Lg

Subsequently, the formation of the NC was carried out using the nanoprecipitation technique. The nanoprecipitation technique (also called the solvent displacement technique) was described for the first time by Fessi et al. [28]. This technique has certain advantages over other encapsulation techniques, namely, (1) good reproducibility, (2) ability to obtain nanoparticle sizes with a narrow distribution, (3) simplicity, (4) ease of scaling, and (5) being environmentally friendly since the use of large amounts of toxic solvents is limited. Due to the above, nanoprecipitation has become an important strategy in the pharmaceutical, agricultural, food, and cosmetic industries [45]. 

Once the NCs were obtained, their physicochemical characterization was carried out. Their size, polydispersity index (PI), and zeta potential are shown in Table 3. A particle size of 287 ± 5.11 nm was obtained, which is close to the desired size of 200 nm. This nanometric size of NCs allows the increase in the ratio between the surface area and the contact area [46]. That is, the nanometric size would allow for the greater surface area of the NCs to be directly in contact with the surface of the fruit and vegetable product, from which the subsequent release of the active components of EO-*Lg* would occur. In previous studies, poly (lactic acid) nanocapsules with lemongrass EO, with a particle size of 96.4 nm, were applied to apples, and it was found that these had rot lesions three times smaller than those treated with non-encapsulated EO and the control [47]. Likewise, in another study, edible coatings for avocados were made from chitosan nanoparticles (355 ± 25.30 nm) loaded with *Schinus molle* EO, and it was found that this reduced weight and firmness losses [48]. 

**Figure 2:**
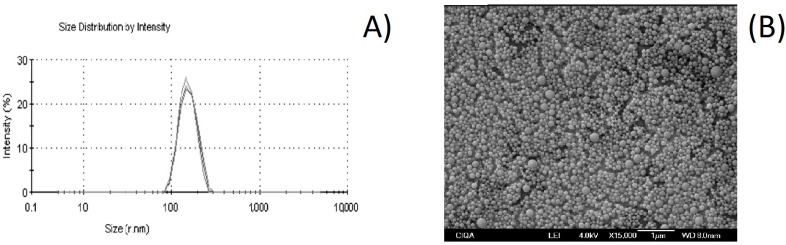
Size evaluation of essential oil *Lippia graveolens* nanocapsules obtained using the nanoprecipitation technique: (**A**) dynamic light scattering analysis and (**B**) scanning electron microscope image.

In relation to the PI, this parameter is associated with the measurement of the degree of variability in the size of the NC. The PI values vary from 0 to 1. The highest value indicates a less homogeneous size distribution, while values close to zero indicate that the sample is monodispersed; that is, it presents minimal variability in population size [49]. A PI of 0.10 ± 0.03 was obtained (Table 3), which indicates a homogeneity of the NCs, and this is also observed in the size distribution curve in Figure 2A. The homogeneity in the size of the NC is a characteristic that would guarantee that individual interactions of the NCs with the surface of the horticultural products (i.e., adhesion or deposition) are also homogeneous. 

The SEM image presented in Figure 2B shows the presence of NCs with a spherical form. There are no significant particle aggregates, and the particle size value (in the 200 nm range) is close to that observed in the DLS analysis (287 ± 5.11 nm). This coincides with a previous study in which chitosan and cashew gum NPs loaded with EO from a species of *Lippia* with a spherical shape were obtained [50]. Likewise, in another study, polycaprolactone NCs loaded with EO from a species of *Lippia* were prepared, in which the morphology of the NCs was spherical, as in the current work [51]. 

Another physicochemical characteristic evaluated was the zeta potential. NCs with a zeta potential greater than +30 mV or less than −30 mV are considered strongly cationic or anionic, respectively, and typically have high degrees of stability [25]. Furthermore, the measurement of the zeta potential ensures that there will be greater separation distances between the NCs in the suspension, which is because the occasional aggregation caused by van der Waals interactions is reduced. These interactions are a consequence of the electrostatic repulsion forces between the NCs. The zeta potential also determines whether a charged active is encapsulated in the center of the NC or on the surface [52]. In this study, the zeta potential of the NCs was −50.90 ± 1.44 mV; this negative value indicates that negative charges are dominant on the surface of the NCs, and this can be attributed to Eudragit L100-55, which is an anionic copolymer. This negative potential would facilitate the interaction of NCs with the membranes of pathogenic bacteria present in fruit and vegetable products, such as strawberries. Subsequently, the direct interaction of the active components of EO-*Lg* with the bacteria would be favored, thus increasing its antimicrobial effectiveness.

On the other hand, as part of the physicochemical characterization of the NCs, the encapsulation percentage (%E) and the encapsulation efficiency percentage (%EE) of the three majority components were also determined: myrcene, p-cymene, and carvacrol. These parameters allow the establishment of the content of the EO components in the NCs in terms of their correct dosage in the fruit and vegetable product. To quantify the three majority components of EO-*Lg* in the NCs, *HS*-SPME validated via the GC-FID method was used. Table 3 shows the %E and %EE of the three components of EO-*Lg* in the NCs purified through evaporation under reduced pressure.

A %E of 0.10 was obtained for myrcene, 0.17 for p-cymene, and 27.91% for carvacrol, and the above represents a total %E of 28.18%. This means that approximately 30% of an NC is formed through these three components of EO-*Lg*, and the remaining 70% corresponds to the NC-forming polymer. It should be noted that Salas-Cedillo [53] used the same polymer as was used in this study to encapsulate the *Schinus molle* EO using the nanoprecipitation technique and obtained a total %E of 7.84% for the three majority components (myrcene, α-phellandrene, and limonene). This value is almost three times smaller than the one obtained in this study. This could be because the encapsulation depends on the physicochemical properties of the components (i.e., polarity and volatility) as well as the % abundance in the EO. 

The %EEs obtained from the NCs were 0.24, 0.32, and 63.80% for myrcene, p-cymene, and carvacrol, respectively; thus, a total %EE of 64.36% was obtained. The above indicates that the three components were encapsulated by approximately 65% in comparison with their abundance in the initial EO. This is similar to what was reported by Pinto et al. [51], who obtained an %EE of 60% when they encapsulated EO from a species of *Lippia* in NPs with chitosan and 1% cashew gum. Likewise, this is similar to what was reported in other publications, which mention that the encapsulation efficiency percentages of non-polar active ingredients are around 80% when the nanoprecipitation technique is used [54]. This could be because the polar components have a greater tendency to diffuse from the organic phase to the aqueous phase, which could favor the encapsulation of non-polar compounds.

### 3.5. Preparation of the Coating 

**Figure 3:**
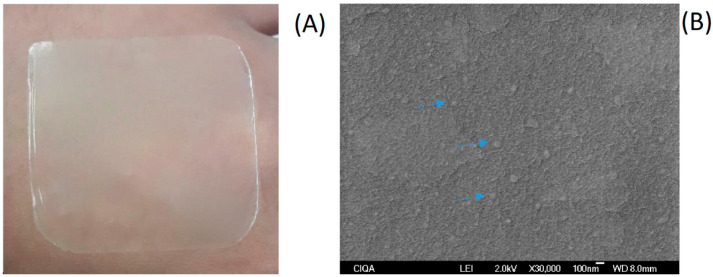
Multisystem coating based on NC-EO-*Lg*-AL formed through the casting method (**A**); NC-EO-*Lg*-AL multisystem coating scanning electron microscope image (**B**) Blue arrows indicate particles of approximately 200 nm homogeneously distributed in the coating.

Once the NCs were obtained and characterized, the formation of the multisystem coating was carried out through the conventional casting method. This technique involves obtaining the dispersion of the coating components (NC and AL) and the evaporation of the solvent or water at a controlled temperature and humidity to form the multisystem coating [55]. For the formation of the multisystem coating, a drying time of 24 h and an AL concentration of 1% *w*/*v* were used. Macroscopic visual and microscopic evaluation of the multisystem coating were carried out. These physical properties of the multisystem coating are fundamental since they influence the appearance of the fruit and vegetable product once the coating is applied. Figure 3A shows that the multisystem coating obtained was homogeneous and completely formed.

SEM analysis is commonly used for the description of packaging surfaces, describing their homogeneity, integrity, smoothness, and the presence of cracks, which can influence the mechanical properties of multisystem coatings. Figure 3B shows that the NC-EO-*Lg*-AL exhibited a soft, uniform, and continuous surface, indicating homogeneity and structural integrity. This could be due to the fact that the presence of the phenolic components of EO-*Lg* in the NC of the multisystem coating acts as crosslinkers and, therefore, results in more uniform multisystem coating [56].

The SEM image shows a coating without cracks as well as particles of approximately 200 nm (indicated by blue arrows) homogeneously distributed. It has been reported that the presence of NC-EO favors the formation of homogeneous coatings [57]. However, previous studies also report that high amounts of EO in NCs generate thicker and more heterogeneous coatings [58]. It is worth mentioning that the amount of the EO-*Lg* encapsulated in polymeric NCs in this study favored the homogeneity of the coating.

### 3.6. Physical Characterization of the Coatings

#### 3.6.1. Optical Evaluation

The optical properties of multisystem coatings are an essential sensory aspect for their acceptance by consumers. They are generally expected to be transparent, similar to polymeric packaging materials, or close in color to the food to which the coating will be applied [59]. In this analysis, higher values indicate less transparency and more opacity. Table 4 shows the results of the optical evaluation. It should be noted that all the multisystem coatings were clear enough to be used as see-through packaging. The AL multisystem coating showed lower opacity (higher transparency) than the NC-BCO-AL multisystem coating. This coincides with a previous study in which it was found that when adding a nanosystem (nanofibers), the opacity increased. This is possibly due to the scattering of light by nanometer-sized particles [60]. However, a decrease in opacity was observed due to the incorporation of the NC-EO-*Lg* compared with the multisystem coating from AL and NP-BCO-AL (nanoparticles without EO-*Lg*). It should be noted that the opacities of the AL and NP-BCO-AL coatings showed significant differences (*p* < 0.05) with respect to the multisystem coating’s opacity. These results are similar to those obtained by Bathia et al. [56], who formed gelatin-based (porcine and bovine) edible films loaded with spearmint EO. They observed that gelatin-based films with the EO showed a decrease in opacity compared to control films that did not utilize the EO. However, it is necessary to mention that the mechanism by which EOs in NCs increase transparency is not entirely clear; in fact, there are various instances where EOs decrease transparency [58].

#### 3.6.2. Mechanical Evaluation

The evaluation of mechanical parameters such as adhesion, tensile strength, and elongation at break will allow the verification of the characteristics of the related coatings from the preparation stage to their application. A coating must possess the following: (i) a good percentage of elongation in order to avoid breaking during packaging or handling; (ii) adequate adhesion to the surfaces of the fruit and vegetable products onto which it will be applied; (iii) good resistance to breaking to ensure integrity; and (iv) a soft, smooth, and transparent texture to not compromise physical appearance. 

The adhesion indicates the magnitude of the force that has to be applied to the multisystem coating to be detached from the surface where it was placed. The force that must be applied to the multisystem coating-based NC-EO-*Lg*-AL for detachment was 5802.36 ± 2.15 dynes cm^−2^ (Table 4). This value showed a significant difference (*p* < 0.05) to those of the NP-BCO-AL and AL coatings. That is, the NC-EO-*Lg* favors the adhesion of the multisystem coating on the applied surface. Likewise, this effect was observed in the study carried out by González-Moreno et al. [24], where the adhesion of multisystem coatings based on NCs loaded with *Thymus vulgaris* EO was evaluated. A value of 4768.27 ± 2.63 dynes cm^−2^ was obtained. The adhesion parameter is indicative of the permanence of the coating on the fruit and vegetable products. The adhesion properties of the coatings are determined by the intrinsic properties of the NC-forming polymer and the environment in which it is placed. Among the intrinsic properties are the molecular weight, the concentration of the polymer, the flexibility of the polymer chains, and the chemical groups that are able to form electrostatic or secondary bonds such as hydrogen bonds and van der Waals forces. 

On the other hand, the tensile strength was also influenced by the presence of the NC-EO-*Lg*. This parameter indicates the weight that the coating can withstand before breaking. In Table 4, it can be observed that the multisystem coating formed from NC-EO-*Lg*-AL can resist 1555.26 ± 4.31 g cm^−2^ before breaking. This value showed a significant difference (*p* < 0.05) to those of NP-BCO-AL and AL coating. That is, EO-*Lg* incorporated into NCs increases the resistance of multisystem coating when a force is applied. The increase in breaking strength by the EOs was also observed in the study developed by Jancy et al. [61], who utilized a multisystem coating-based NP-EO derived from fennel seed. It was found that the presence of the NP-EO increased the breaking strength by up to seven times. This can be explained by the results of other studies, which have shown that an NC-EO is able to increase the tensile strength of multisystem coatings, probably due to cross-linking processes. In this sense, EOs with phenolic compounds, such as the EO-*Lg*, can act as cross-linkers in multisystem coatings [62]. 

The percentage of elongation (%) indicates the maximum percentage change in the length of the multisystem coating before breaking [63]. Table 4 shows that the NC-EO-*Lg*-AL multisystem coating can be stretched or elongated by 182.27 ± 2.14% of its initial size. This value showed a significant difference (*p* < 0.05) to those of NP-BCO and AL coatings. This characteristic of increasing stretch is attributed to the presence of NC-EO-*Lg* in the multisystem coating. The effect of the addition of the NC-EO on the mechanical properties of multisystem coatings is quite complex, and the improvement in stretching or elongation characteristics has been reported in the literature. For instance, in the research carried out by Liang et al. [64], multisystem films based on chitosan and NCs loaded with epigallocatechin gallate were developed. The addition of these NCs to chitosan multisystem films increased the percentage of elongation in comparison to the edible films formed only with chitosan. Likewise, similar findings were reported by Noronha et al. [65], who obtained a multisystem coating from methylcellulose with NC-α-tocopherol. Significant changes in the percentage of elongation were reported with the incorporation of NCs into multisystem coatings compared to the control coating. The increase in the elongation percentage values indicated that the incorporation of NCs provides greater flexibility in the coatings. This could indicate that the addition of NCs would also modify the interactions with coating-forming agents such as AL. It has been proposed that a homogeneous dispersion of hydrophobic NC in an AL polymer network increases the spacing between the chains of macromolecules, which reduces ionic and hydrogen bonding between the polymer chains and induces the development of structural discontinuities in the chains of the multisystem coatings, which would increase the percentage of elongation [66].

It should be noted that the mechanical evaluation of the multisystem coating will depend on the type of material used in the formulation of the solution to make the multisystem coating, the conditions of formation, the type of plasticizer, the nature of the solvent, the evaporation of the solvent, and the thickness. It is worth highlighting that depending on use and application, the desired physical and mechanical properties will change in order to meet the objectives for which multisystem coatings were created.

#### 3.6.3. Fourier-Transform Infrared (FTIR) Spectroscopy Analysis

**Figure 4:**
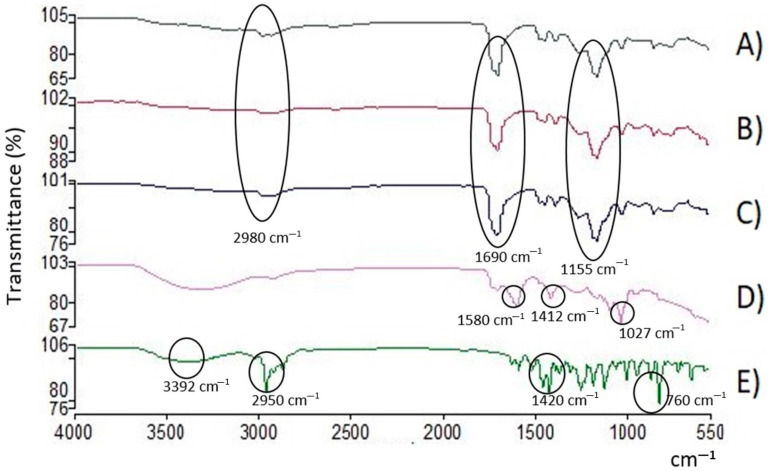
FT-IR spectrum of (**A**) polymer, (**B**) NP-BCO, (**C**) NC-EO-*Lg*, (**D**) NC-EO-*Lg*-AL, and (**E**) EO-*Lg*. The characteristic bands of the functional groups are indicated.

An analysis via infrared spectroscopy was carried out in order to establish the interactions between the components of the coatings. Each of the components was analyzed separately: (A) the Eudragit L100-55 polymer, (B) the NP-BCO, (C) the NC-EO-*Lg*, (D) the NC-EO-*Lg*-AL, and (E) the free EO-*Lg*. The spectra are shown below in Figure 4.

FT-IR spectroscopy is used to identify functional groups and chemical interactions between formulation components. The middle region of the infrared spectrum (400–4000 cm^−1^) is widely used to study fundamental vibrations and the rotational–vibrational structure associated with different chemical bonds. Figure 4A shows the spectrum of the Eudragit L100-55 polymer, in which four important signals of the main chemical groups present in the polymer unit of this copolymer can be identified. In the region between 2500 and 3500 cm^−1^, the stretching of the hydroxyl group (O–H) belonging to the carboxylic acid present in the side chain of the polymer unit can be observed, and in the region of 2980 cm^−1^, the C–H signals can be observed. In the region of 1690 cm^−1^, stretching, which corresponds to the carbonyl groups (C=O) present in the side chains of the carboxylic acid and the ester, can be observed. Finally, in the region of 1155 cm^−1^, the stretching corresponding to the C–O–C group present in the side chain of the ester can be observed. The aforementioned signals coincide with what was reported in the technical sheet of the Eudragit L100-55 polymer. Likewise, in Figure 4B,C, the IR spectra obtained from the NP-BCO and the NC-EO are observed, respectively. In both spectra, it can be seen that the signals obtained correspond mainly to the signals obtained in the polymer spectrum (Figure 4A). Therefore, no signals are attributed to the spectrum of the EO-*Lg*. Regarding the NC-EO-*Lg*, it can be inferred that the oil components are encapsulated in the framework of the NC and are not in a free or a superficial form. It is important to highlight that the signals with greater intensity correspond to the majority components of the EO-*Lg*. On the other hand, Figure 4D shows the spectrum obtained for the NC-EO-*Lg*-AL. It can be seen that the signals obtained correspond mainly to the AL, which could indicate that at the time of adding AL to the NC suspension, the chains of this polysaccharide were organized on the polymeric wall of the NC. And, since no signals attributed to the spectrum of the EO-*Lg* were observed, it is inferred that, as in the NC-EO-*Lg*, the oil components are encapsulated in the NC and are not in free form. These results showed that EO-*Lg* was encapsulated in the NC without changing its structure or function, which coincides with what was reported by Ma et al. [67]. Finally, in Figure 4E, the IR spectrum obtained from the EO-*Lg* is shown, where a band of 3392 cm^−1^ can be seen that belongs to the O–H bond vibrations characteristics of the phenolic components present in EO, such as carvacrol and thymol. Another observable band is that of 2950 cm^−1^, which appears due to the presence of the CH_3_ or CH_2_ bonds of the methyl and methylene groups that are frequently found in the structures of organic compounds. Within the region of 1101–1209 cm^−1^, characteristic bands of the C–OH bonds of primary and secondary alcohols are present in molecules such as terpinen-4-ol and 1,8-cineole, which agrees with what was reported by Matadamas-Ortiz et al. [14]. In the region of 850–1080 cm^−1^, bands belonging to the C–O groups are observable. 

#### 3.6.4. Water Vapor Barrier Properties 

An important property of coatings is their ability to prevent moisture exchange between the medium and the interior of the food. WVTR is the measure of the moisture that moves through a unit space of material per unit time [68]. On the other hand, WVP is a vital property used to evaluate the ability of films to act as a moisture barrier. The results obtained for WVTR and WVP are presented in Table 4. The NC-EO-*Lg*-AL multisystem coating performed better as a moisture barrier compared to the AL and NP-BCO-AL coatings because it presented lower WVTR and WVP values with significant differences (*p* < 0.05). This decrease in the WVTR and WVP values could be due to the fact that the presence of nanoencapsulated EO-*Lg* modifies the hydrophilic/hydrophobic ratio of the coating, thus affecting water transmission through the hydrophilic part of this coating. Lower WVTR and WVP values are preferred since this reduces the moisture loss of wrapped food products [69]. This agrees with the results reported by Al-Harrasi et al. [70], who reported that multisystem gelatin–sodium alginate films loaded with ginger EO decrease WVP values.

Therefore, the EO-*Lg* incorporated into the NCs acted as a plasticizing agent in the coating, favoring the accommodation of the NCs in the epicarp and strawberry seeds, thus reducing water loss. Thus, the hydrophobic bioactive substances that increase the hydrophobicity of the multisystem coatings reduce water vapor migration. 

### 3.7. Application of Multisystem Coatings in Strawberries

#### 3.7.1. Weight Loss Percentage and Texture Analyses: Penetration Test

**Figure 5:**
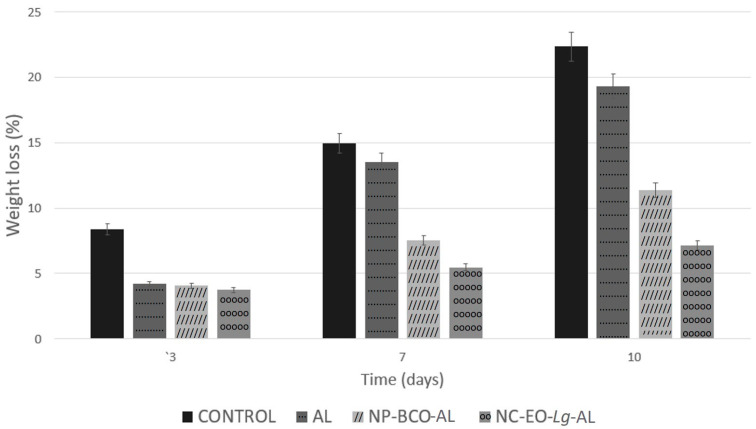
Weight loss percentages of strawberry fruits coated with alginate, NP-BCO-AL, or NC-EO-*Lg*-AL and the control (without multisystem coating) during storage at 4 °C (mean ± SD, *n* = 7).

To determine the effectiveness of the multisystem antimicrobial coating in improving the quality of fresh strawberries, the percentage of weight loss was utilized as a quality marker during storage time and compared with strawberries without a coating (control). The results show that the percentage of weight loss increased during storage time in all strawberries (Figure 5). The weight loss in the untreated strawberries (control) was much greater than in the strawberries coated with a multisystem coating.

Overall, based on Figure 5, the percentage of weight loss in the control strawberries on the 10th observation day was 22.35%, while in the strawberries coated with NC-EO-*Lg*-AL, the weight loss was 7.12%. The weight loss percentage value showed significant differences (*p* < 0.05) in strawberries with the multisystem coating (NC-EO-*Lg*-AL) in comparison to strawberries with the NP-BCO-AL and AL coatings. These results coincide with those reported by Martínez et al. [71], who developed and applied multisystem chitosan-based coatings with *Thymus capitatus* EO to strawberries. In this study, less weight loss was observed in strawberries that had the chitosan coating and EO in comparison to strawberries that did not have a coating and those that had the chitosan-based coating without the EO. The results of this study support the advantages of applying multisystem coating to strawberries since it has been shown to prevent water loss from freshly cut strawberries.

Weight loss is commonly attributed entirely to water loss [72]. Strawberries are highly perishable, so they are easily susceptible to water loss, which results in the weakening of the fruit tissue due to their very thin epicarp. This negatively affects the appearance of the fruit, causing softening, the development of pathogens, and wrinkling, as well as a change in color and aroma, which accelerates senescence and subsequently causes serious economic losses. Multisystem coatings with NC-EO-*Lg*-AL provide a barrier function, protecting the fruit from the outside, in addition to also limiting transpiration, which delays dehydration [73]. On the other hand, the fruit firmness of the coated strawberries during the storage time was analyzed because this represents one of the essential parameters for determining fruit quality. Strawberries have a very active metabolism and are subject to mechanical damage, attacks by microorganisms, and loss of quality during storage. The decrease in firmness is due to the greater loss of water vapor from the surface of the fruit to the outside atmosphere, which can encourage the growth of fungi and bacteria. These microorganisms cause structural damage to tissues and allow them to soften. Furthermore, the decrease in firmness is related to the increase in moisture loss. It should be noted that the decrease in firmness could be related to the degradation of the cell wall, specifically the cortical parenchyma, as a consequence of the enzymatic degradation processes and the loss of moisture during the storage period.

**Figure 6:**
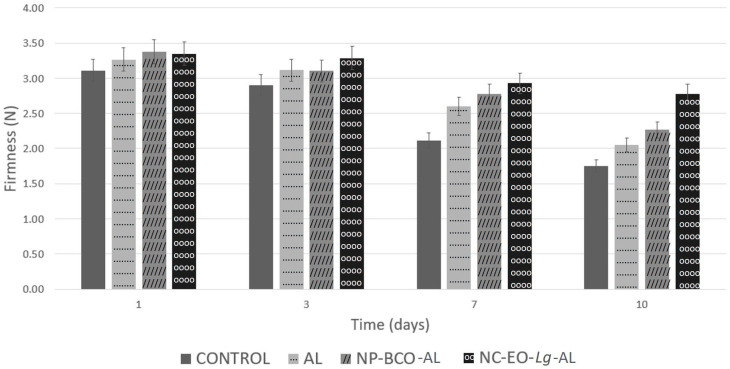
Firmness of strawberries coated with alginate, NP-BCO-AL, or NC-EO-*Lg*-AL and the control (without multisystem coating) after storage for 10 days at 4 °C (mean ± SD, *n* = 7).

Overall, based on Figure 6, at time 0, the firmness of the fruit was 3.11 N, while after ten storage days, decreased firmness was observed, both in the uncoated and coated samples, but the lowest value was found in the control strawberries (1.92 N). In percentage terms, after 10 days of cold storage, the control group lost 43.64% of firmness, while the AL and NP-BCO-AL coating treatment only lost 37.16 and 32.8%, respectively. On the other hand, the highest firmness values were recorded in strawberries with an NC-EO-*Lg*-AL multisystem coating (2.78 N), which lost only 17.02% firmness. It should be noted that the firmness value of the multisystem coating showed significant differences (*p* < 0.05) in comparison to the AL and NP-BCO-AL coatings. The results of this study coincide with those reported by De-Bruno et al. [74], who applied edible coating enriched with bergamot EO to strawberries and obtained a lower loss of firmness compared to the control group. Furthermore, it can be inferred that in this study, the NC-EO-*Lg*-AL multisystem coating had the ability to act as a barrier that interferes with gas exchange, which leads to a reduction in the respiration rate of the fruit and prevents water loss, as observed in Table 4; furthermore, it is possible that the antioxidant activity of the EO-*Lg* components decreased the enzymatic activity of the fruit, resulting in the slower ripening of the strawberry.

#### 3.7.2. Visual Microbiological Damage

**Figure 7:**
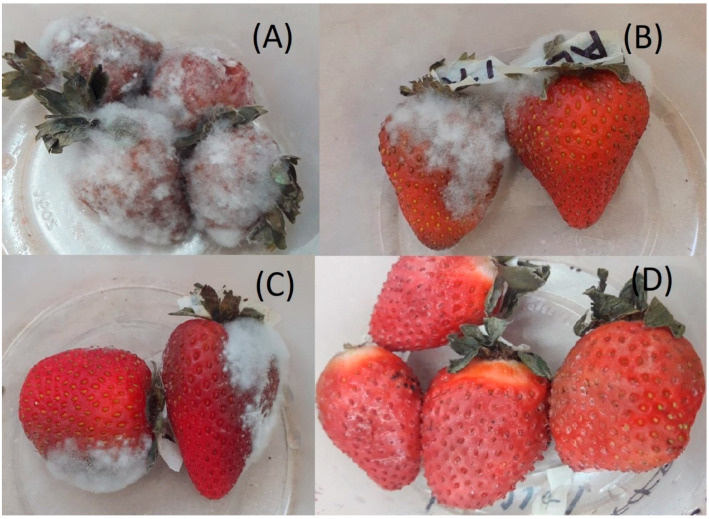
Visual microbiological damage of strawberries after 15 days of storage at 4 °C: uncoated (control) (**A**), AL coating (**B**), NP-BCO coating (**C**), and NC-EO-*Lg* multisystem coating (**D**).

Regarding visual microbiological damage, fungal growth on the surface of the strawberry began to be observed rapidly in the control samples after 7 days of storage at 4 °C. After 15 days, they already showed considerable microbiological damage (Figure 7A). Likewise, the strawberries coated with the AL (Figure 7B) and NP-BCO-AL coating (Figure 7C) also showed microbiological damage after storage time. On the other hand, the strawberries coated with NC-EO-*Lg*-AL multisystem coating did not present microbiological damage (Figure 7D). 

It should be noted that strawberries with the NC-EO-*Lg*-AL multisystem coating were the most efficient in reducing microbial decomposition and could be considered an effective treatment to reduce the deterioration of fresh strawberries during their storage period. The growth of pathogens such as fungi and bacteria on the surface of strawberries in refrigerated conditions is a serious problem that reduces the number of purchases by consumers, so the multisystem coatings used in this study are a good alternative to increase shelf life in refrigerated conditions. Different studies have been carried out using EO coatings in order to control the decomposition of fungi and bacteria and extend the shelf life of fruits and vegetables [75]. 

## 4. Conclusions

Smart biodegradable and multisystem coatings may be good alternative candidates for the postharvest preservation of fruits, especially strawberries, which suffer a rapid decrease in quality due to their high metabolism and rapid development of pathogens on their surface. Our observations in this study demonstrated that multisystem coating fruit with NC-EO-*Lg* decreased the percentage of weight loss and loss of firmness of strawberries, which is why it is advisable to use this to maintain the quality of strawberries during storage life and marketing periods. Thus, it is considered to be a safe and effective method as well as an environmentally friendly technology, and it also has the advantage of being a method that can be reproduced on an industrial scale.

## Figures and Tables

**Table 1:** Physical characterization of anethole control and essential oil of *Lippia graveolens*.

Parameter	Relative Density ^1^	Refractive Index (°) ^1^	Specific Rotation (g/mL) ^2^
Anethole (FEUM)	0.983 − 0.988	1.557 − 1.561	−0.150 − 0.150
Anethole	0.987 ± 0.000	1.559 ± 0.000	0.050 ± 0.000
EO-*Lippia graveolens*	0.987 ± 0.000	1.503 ± 0.000	−0.200 ± 0.000
EO-*Lippia alba*	0.945 ± 0.005	1.462 ± 0.000	−0.117 ± 0.028

^1^ ($\bar{x}$ ± σ, *n* = 3); ^2^ ($\bar{x}$ ± σ, *n* = 6).

**Table 2:** Chemical composition of essential oil of *Lippia graveolens* by gas chromatography–mass spectrometry.

No. ^1^	Composition	tR (min) ^2^	Abundance	Component Type
1	α-thujene	16.51	0.19	Monoterpene hydrocarbon
2	α-pinene	17.83	0.08	Monoterpene hydrocarbon
3	myrcene	18.37	16.93	Monoterpene hydrocarbon
4	α-terpinene	18.92	0.08	Monoterpene hydrocarbon
5	p-cymene	20.25	7.56	Monoterpene hydrocarbon
6	1,8-sineole	22.88	0.04	Monoterpene hydrocarbon
7	γ-terpinene	26.18	0.08	Monoterpene hydrocarbon
8	linalool	26.63	0.12	Oxygenated monoterpene
9	terpinen-4-ol	29.29	0.10	Oxygenated monoterpene
10	thymol methyl ether	30.94	0.06	Oxygenated monoterpene
11	thymol	32.49	4.91	Oxygenated monoterpene
12	carvacrol	33.02	66.58	Oxygenated monoterpene
13	z-caryophyllene	36.96	2.99	Sesquiterpene hydrocarbon
14	α-humulene	38.33	0.11	Sesquiterpene hydrocarbon
15	butyl hydroxyanisole	41.49	0.07	Sesquiterpene oxygenated
16	caryophyllene oxide	43.52	0.03	Sesquiterpene oxygenated
	TOTAL		100.00	

^1^ Elution order; ^2^ retention time.

**Table 3:** Content of myrcene, p-cymene, and carvacrol in nanocapsules of *Lippia graveolens* essential oil (NC-EO-*Lg*) purified through evaporation under reduced pressure.

	Mean Size (nm) ^1^	PI ^1^	Zeta Potential (mV) ^1^	Component	%E ^1^	%EE ^1^
NC-EO-*Lg*	287 ± 5.11	0.10 ± 0.03	−50.90 ± 1.44	Myrcene	0.10 ± 0.04	0.24 ± 0.02
				p-cymene	0.17 ± 0.07	0.32 ± 0.01
				Carvacrol	27.91 ± 0.59	63.80 ± 1.47

^1^ ($\bar{x}$ ± σ, *n* = 3).

**Table 4:** Optical, mechanical, and water vapor barrier properties evaluation of the multisystem coating.

Coating	Opacity ^1^ (UA mm^−1^)	Adhesion ^1^ (Dynes cm^−2^)	Tensile Strength ^1^ (g cm^−2^)	Elongation at Break (%) ^1^	WVP ^1^ (10^−7^ g mm cm^−2^ Pa^−1^ h^−1^)	WVTR ^1^ (10^−3^ g cm^−2^ h^−1^)
AL	0.71 ± 0.10	4690.86 ± 2.00	977.56 ± 3.94	90.76 ± 0.71	2.79 ± 0.04	4.68 ± 0.09
NP-BCO-AL	0.96 ± 0.26	4989.22 ± 1.03	575.54 ± 5.27	78.28 ± 0.76	1.97 ± 0.03	3.72 ± 0.08
NC-EO-*Lg*-AL	0.60 ± 0.10	5802.36 ± 2.15	1555.26 ± 4.31	182.27 ± 2.14	1.01 ± 0.03	2.03 ± 0.06

^1^ ($\bar{x}$ ± σ, *n* = 3).

## Data Availability

Data are contained within the article.

## References

[1] FAO (2019). El Estado de la Alimentación y la Agricultura. Avanzar en la Reducción de la Pérdida y el Desperdicio de Alimentos.

[2] Curiel J.A. (2021). Application of biotechnological techniques aimed to obtain bioactive compounds from food industry by-products. Biomolecules.

[3] Pace B., Cefola M. (2021). Innovative preservation technology for the fresh fruit and vegetables. Foods.

[4] Torres-Sánchez R., Martínez-Zafra M.T., Castillejo N., Guillamón-Frutos A., Artés-Hernández F. (2020). Real-time monitoring system for shelf life estimation of fruit and vegetables. Sensors.

[5] Mendoza-Barboza C.R., Borges C.D., Kringel A.L., da-Silveira R.P., da-Silva F.A., Schulz G.A.S. (2020). Application of microemulsions as coating in fresh cut strawberries. J. Food Sci. Technol..

[6] Trinetta V., McDaniel A., Batziakas K., Yucel U., Nwadike L., Pliakoni E. (2020). Antifungal packaging film to maintain quality and control postharvest diseases in strawberries. Antibiotics.

[7] Salsabiela S., Sukma-Sekarina A., Bagus H., Audiensi A., Azizah F., Heristika W., Susanto E., Munawaroh H.S.H., Show P.L., Ningrum A. (2022). Development of edible coating from gelatin composites with the addition of black tea extract (*Camellia sinensis*) on minimally processed watermelon (*Citrullus lanatus*). Polymers.

[8] Paolucci M., Di-Stasio M., Sorrentino A., La-Cara F., Volpe M.G. (2020). Active edible polysaccharide-based coating for preservation of fresh figs (*Ficus carica* L.). Foods.

[9] Nor S., Ding P. (2020). Trends and advances in edible biopolymer coating for tropical fruit: A review. Food Res. Int..

[10] Salas-Méndez E., Pinheiro A.C., Ballesteros L.F., Silva P., Rodríguez-García R., Jasso-de-Rodríguez D. (2019). Application of edible nanolaminate coatings with antimicrobial extract of *Flourensia cernua* to extend the shelf-life of tomato (*Solanum lycopersicum* L.) fruit. Postharvest Biol. Technol..

[11] Yin C., Huang C., Wang J., Liu Y., Lu P., Huang L. (2019). Effect of chitosan and alginate-based coatings enriched with cinnamon essential oil microcapsules to improve the postharvest quality of mangoes. Materials.

[12] Tavares L.S., de-Souza V.C., Schmitz-Nunes V., Nascimento-Silva O., de-Souza G.T., Farinazzo-Marques L., Capriles-Goliatt P.V.Z., Facio-Viccini L., Franco O.L., de-Oliveira-Santos M. (2020). Antimicrobial peptide selection from *Lippia* spp leaf transcriptomes. Peptides.

[13] Gutiérrez-Grijalva E.P., Antunes-Ricardo M., Acosta-Estrada B.A., Gutiérrez-Uribe J.A., Heredia J.B. (2019). Cellular antioxidant activity and in vitro inhibition of α-glucosidase, α- amylase and pancreatic lipase of oregano polyphenols under simulated gastrointestinal digestion. Int. Food Res. J..

[14] Matadamas-Ortiz A., Hernández-Hernández E., Castaño-Tostado E., Amaro-Reyes A., García-Almendárez B.E., Velazquez G., Regalado-González C. (2022). Long-term refrigerated storage of beef using an active edible film reinforced with mesoporous silica nanoparticles containing oregano essential oil (*Lippia graveolens* Kunth). Int. J. Mol. Sci..

[15] Rodríguez-García I., Cruz-Valenzuela M.R., Silva-Espinoza B.A., Gonzalez-Aguilar G.A., Moctezuma E., Gutierrez-Pacheco M.M., Tapia-Rodriguez M.R., Ortega-Ramirez L.A., Ayala-Zavala J.F. (2016). Oregano (*Lippia graveolens*) essential oil added within pectin edible coatings prevents fungal decay and increases the antioxidant capacity of treated tomatoes. J. Sci. Food Agric..

[16] Zhang J., Ma S., Du S., Chen S., Sun H. (2019). Antifungal activity of thymol and carvacrol against postharvest pathogens *Botrytis cinerea*. J. Food Sci. Technol..

[17] Leyva-López N., Gutiérrez-Grijalva E.P., Vazquez-Olivo G., Heredia J.B. (2017). Essential oils of oregano: Biological activity beyond their antimicrobial properties. Molecules.

[18] Herrera-Rodríguez S.E., López-Rivera R.J., García-Márquez E., Estarrón-Espinosa M., Espinosa-Andrews H. (2019). Mexican oregano (*Lippia graveolens*) essential oil-in-water emulsions: Impact of emulsifier type on the antifungal activity of *Candida albicans*. Food Sci. Biotechnol..

[19] Bhargava K., Conti D.S., da-Rocha S.R.P., Zhang Y. (2015). Application of an oregano oil nanoemulsion to the control of foodborne bacteria on fresh lettuce. Food Microbiol..

[20] Asbahani A.E., Miladi K., Badri W., Sala M., Addi E.H.A., Casabianca H., Elaissari A. (2015). Essential oils: From extraction to encapsulation. Int. J. Pharm..

[21] Rai M., Paralikar P., Jogee P., Agarkar G., Ingle A.P., Derita M., Zacchino S. (2017). Synergistic antimicrobial potential of essential oils in combination with nanoparticles: Emerging trends and future perspectives. Int. J. Pharm..

[22] Odetayo T., Tesfay S., Ngobese N.Z. (2022). Nanotechnology-enhanced edible coating application on climacteric fruits. Food Sci. Nutr..

[23] Zhang M., Luo W., Yang K., Li C. (2022). Effects of sodium alginate edible coating with cinnamon essential oil nanocapsules and nisin on quality and shelf life of beef slices during refrigeration. J. Food Prot..

[24] González-Moreno B.J. (2020). Cubierta Biopolimérica de Nanoingredientes a Base de *Thymus vulgaris* con Potencial Aplicación en Productos Hortofrutícolas. Master’s Thesis.

[25] Piña-Barrera A.M., Álvarez-Román R., Báez-González J.G., Amaya-Guerra C.A., Rivas-Morales C., Gallardo-Rivera C.T., Galindo-Rodríguez S.A. (2019). Application of a multisystem coating based on polymeric nanocapsules containing essential oil of *Thymus vulgaris* L. to increase the shelf life of table grapes (*Vitis vinifera* L.). IEEE Trans. Nanotechnol..

[26] Samba N., Aitfella-Lahlou R., Nelo M., Silva L., Coca R., Rocha P., López-Rodilla J.M. (2020). Chemical composition and antibacterial activity of *Lippia multiflora* moldenke essential oil from different regions of Angola. Molecules.

[27] Permanent Commission of Pharmacopoeia of the United Mexican States (2011). Farmacopea de los Estados Unidos Mexicanos.

[28] Fessi H., Puisieux F., Devissaguet J.P., Ammoury N., Benita S. (1989). Nanocapsule formation by interfacial polymer deposition following solvent displacement. Int. J. Pharm..

[29] Ye J., Ma D., Qin W., Liu Y. (2018). Physical and antibacterial properties of sodium alginate-sodium carboxymethylcellulose films containing *Lactococcus lactis*. Molecules.

[30] Bhatia S., Al-Harrasi A., Al-Azri M.S., Ullah S., Bekhit A.E.A., Pratap-Singh A., Chatli M.K., Anwer M.K., Aldawsari M.F. (2022). Preparation and physiochemical characterization of bitter orange oil loaded sodium alginate and casein based edible films. Polymers.

[31] (1995). Standard Test Method for Water Vapor Transmission of Materials.

[32] Siracusa V., Romani S., Gigli M., Mannozzi C., Cecchini J.P., Tylewicz U., Lotti N. (2018). Characterization of active edible films based on citral essential oil, alginate and pectin. Materials.

[33] Dilworth L., Riley C.K., Stennett D. (2017). Plant Constituents: Carbohydrates, oils, resins, balsams, and plant hormones. Pharmacognosy Fundamentals, Applications and Strategie.

[34] Hernández-Hernández E., Regalado-González C., Vázquez-Landaverde P., Guerrero-Legarreta I., García-Almendárez B.E. (2014). Microencapsulation, chemical characterization, and antimicrobial activity of Mexican (*Lippia graveolens* H.B.K.) and European (*Origanum vulgare* L.) oregano essential oils. Sci. World J..

[35] Lugo-Estrada L., Galindo-Rodríguez S.A., Pérez-López L.A., Waksman-deTorres N., Álvarez-Román R. (2019). Headspace–solid-phase microextraction gas chromatography method to quantify *Thymus vulgaris* essential oil in polymeric nanoparticles. Pharmacogn. Mag..

[36] Torrenegra-Alarcón M.E., Matiz-Melo G.E., González J.G., León-Méndez G. (2015). In vitro antibacterial activity of the essential oil against microorganisms involved in acné. Rev. Cuba. Farm.

[37] Domínguez X. (1985). Métodos de Investigación Fitoquímica.

[38] Adams R.P. (2007). Identification of Essential Oil Components by Gas Chromatography/Mass Spectrometry.

[39] Chacón-Vargas K.F., Sánchez-Torres L.E., Chávez-González M.L., Adame-Gallegos J.R., Nevárez-Moorillón G.V. (2022). Mexican oregano (*Lippia berlandieri* Schauer and *Poliomintha longiflora* Gray) essential oils induce cell death by apoptosis in *Leishmania* (leishmania) *mexicana* promastigotes. Molecules.

[40] Medina-Romero Y.M., Rodriguez-Canales M., Rodriguez-Monroy M.A., Hernandez-Hernandez A.B., Delgado-Buenrostro N.L., Chirino Y.I., Cruz-Sanchez T., Garcia-Tova C.G., Canales-Martinez M.M. (2022). Effect of the essential oils of *Bursera morelensis* and *Lippia graveolens* and five pure compounds on the mycelium, spore production, and germination of species of fusarium. J. Fungi.

[41] Capatina L., Napoli E.M., Ruberto G., Hritcu L. (2021). *Origanum vulgare* ssp. hirtum (Lamiaceae) essential oil prevents behavioral and oxidative stress changes in the scopolamine zebrafish model. Molecules.

[42] Sharififard M., Alizadeh I., Jahanifard E., Wang C., Azemi M.E. (2018). Chemical composition and repellency of *Origanum vulgare* essential oil against cimex lectularius under laboratory conditions. J. Arthropod. Borne Dis..

[43] Ribeiro F.P., Santana-de-Oliveira M., de-Oliveira-Feitosa A., Santana-Barbosa- Marinho P., Moacir-do-Rosario-Marinho A., de-Aguiar-Andrade E.H., Favacho-Ribeiro A. (2021). Chemical composition and antibacterial activity of the *Lippia origanoides* Kunth essential oil from the Carajás National Forest, Brazil. Evid. Based Complement. Alternat. Med..

[44] Amiri H. (2012). Essential oils composition and antioxidant properties of three thymus species. Evid.-Based Complement. Altern. Med..

[45] Miladi K., Sfar S., Fessi H., Elaissari A. (2015). Encapsulation of alendronate sodium by nanoprecipitation and double emulsion: From preparation to in vitro studies. Ind. Crops. Prod..

[46] Sotelo-Boyás M.E., Correa-Pacheco Z.N., Bautista-Baños S., Corona-Rangel M.L. (2017). Physicochemical characterization of chitosan nanoparticles and nanocapsules incorporated with lime essential oil and their antibacterial activity against food-borne pathogens. Food Sci. Technol..

[47] Antonioli G., Fontanella G., Echeverrigaray S., Longaray-Delamare A.P., Fernandes-Pauletti G., Barcellos T. (2020). Poly(lactic acid) nanocapsules containing lemongrass essential oil for postharvest decay control: In vitro and in vivo evaluation against phytopathogenic fungi. Food Chem..

[48] Chávez-Magdaleno M.E., González-Estrada R.R., Ramos-Guerrero A., Plascencia-Jatomea M., Gutiérrez-Martínez P. (2018). Effect of pepper tree (*Schinus molle*) essential oil-loaded chitosan bio-nanocomposites on postharvest control of *Colletotrichum gloeosporioides* and quality evaluations in avocado (*Persea americana*) cv. Hass. Food Sci. Biotechnol..

[49] Galindo-Rodríguez S.A., Allemann E., Fessi H., Doelker E. (2004). Physicochemical parameters associated with nanoparticle formation in the salting-out, emulsification-difussion, and nanoprecipitation methods. Pharm. Res..

[50] Abreu F.O., Oliveira E.F., Paula H.C., de-Paula R.C. (2012). Chitosan/cashew gum nanogels for essential oil encapsulation. Carbohydr. Polym..

[51] Pinto N., Rodrigues T.H.S., Pereira R., Silva L.M.A., Cáceres C.A., Azeredo H.M., de-Canuto K.M. (2016). Production and physico-chemical characterization of nanocapsules of the essential oil from *Lippia sidoides* Cham. Ind Crops Prod..

[52] Fraj A., Jaâfar F., Marti M., Coderch L., Ladhari N. (2019). A comparative study of oregano (*Origanum vulgare* L.) essential oil-based polycaprolactone nanocapsules/microspheres: Preparation, physicochemical characterization, and storage stability. Ind. Crops Prod..

[53] Salas-Cedillo H.I. (2016). Desarrollo de un Potencial Insecticida Nanoparticulado de *Schinus molle* Para el Control de *Aedes aegypti*. Master’s Thesis.

[54] Cartaxo A.L., Costa-Pinto A.R., Martins A., Faria S., Gonçalves V.M.F., Tiritan M.E., Ferreira H., Neves N.M. (2019). Influence of PDLA nanoparticles size on drug release and interaction with cells. J. Biomed. Mater. Res. A..

[55] Solano-Doblado L.G., Alamilla-Beltrán L., Jiménez-Martínez C. (2018). Películas y recubrimientos comestibles funcionalizados. Rev. Espec. Cienc. Químico-Biológicas.

[56] Bhatia S., Al-Harrasi A., Shah Y.A., Jawad M., Al-Azri M.S., Ullah S., Anwer M.K., Aldawsari M.F., Koca E., Aydemir L.Y. (2023). Physicochemical characterization and antioxidant properties of chitosan and sodium alginate based films incorporated with ficus extract. Polymers.

[57] Yadav R.K., Nandy B.C., Maity S., Sarkar S., Saha S. (2015). Phytochemistry, pharmacology, toxicology, and clinical trial of *Ficus racemosa*. Pharmacogn. Rev..

[58] Ma Y., Chen S., Liu P., He Y., Chen F., Cai Y., Yang X. (2023). Gelatin improves the performance of oregano essential oil nanoparticle composite films-application to the preservation of mullet. Foods.

[59] Galus S., Kadzińska J. (2016). Moisture sensitivity, optical, mechanical and structural properties of whey protein-based edible films incorporated with rapeseed oil. Food Technol. Biotechnol..

[60] Medina-Jaramillo C., Quintero-Pimiento C., Gómez-Hoyos C., Zuluaga-Gallego R., López-Córdoba A. (2020). Alginate-edible coatings for application on wild andean blueberries (*Vaccinium meridionale* Swartz): Effect of the addition of nanofibrils isolated from cocoa by-products. Polymers.

[61] Jancy S., Shruthy R., Preetha R. (2020). Fabrication of packaging film reinforced with cellulose nanoparticles synthesised from jack fruit non-edible part using response surface methodology. Int. J. Biol. Macromol..

[62] Xue Y., Lofland S., Hu X. (2019). Thermal conductivity of protein-based materials: A review. Polymers.

[63] Rangel-Marrón M., Mani-López E., Palou E., López-Malo A. (2018). Effects of alginate-glycerol-citric acid concentrations on selected physical, mechanical, and barrier properties of papaya puree-based edible films and coatings, as evaluated by response surface methodology. LWT.

[64] Liang J., Yan H., Wang X., Zhou Y., Gao X., Puligundla P., Wan X. (2017). Encapsulation of epigallocatechin gallate in zein/chitosan nanoparticles for controlled applications in food systems. Food Chem..

[65] Noronha C.M., de-Carvalho S.M., Lino R.C., Barreto P.L.M. (2014). Characterization of antioxidant methylcellulose film incorporated with α-tocopherol nanocapsules. Food Chem..

[66] Sánchez-González L., Vargas M., González-Martínez C., Chiralt A., Cháfer M. (2009). Characterization of edible films based on hydroxypropylmethylcellulose and tea tree essential oil. Food Hydrocoll..

[67] Ma Y., Liu P., Ye K., He Y., Chen S., Yuan A., Chen F., Yang W. (2022). Preparation, characterization, in vitro release, and antibacterial activity of oregano essential oil chitosan nanoparticles. Foods.

[68] González-Sandoval D.C., Luna-Sosa B., Martínez-Ávila G.C.G., Rodríguez-Fuentes H., Avendaño-Abarca V.H., Rojas R. (2019). Formulation and characterization of edible films based on organic mucilage from mexican opuntia ficus-indica. Coatings.

[69] Li Y., Yao M., Liang C., Zhao H., Liu Y., Zong Y. (2022). Hemicellulose and nano/microfibrils improving the pliability and hydrophobic properties of cellulose film by interstitial filling and forming micro/nanostructure. Polymers.

[70] Al-Harrasi A., Bhatia S., Al-Azri M.S., Ullah S., Najmi A., Albratty M., Meraya A.M., Mohan S., Aldawsari M.F. (2022). Effect of drying temperature on physical, chemical, and antioxidant properties of ginger oil loaded gelatin-sodium alginate edible films. Membranes.

[71] Martínez K., Ortiz M., Albis A., Gilma-Gutiérrez-Castañeda C., Valencia M.E., Grande-Tovar C.D. (2018). The Effect of edible chitosan coatings incorporated with *Thymus capitatus* essential oil on the shelf-life of strawberry (*Fragaria ananassa*) during cold storage. Biomolecules.

[72] Heristika W., Ningrum A., Supriyadi-Munawaroh H.S.H., Show P.L. (2023). Development of composite edible coating from gelatin-pectin incorporated garlic essential oil on physicochemical characteristics of red chili (*Capsicum annnum* L.). Gels.

[73] Shiekh K.A., Ngiwngam K., Tongdeesoontorn W. (2022). Polysaccharide-Based Active coatings incorporated with bioactive compounds for reducing postharvest losses of fresh fruits. Coatings.

[74] De Bruno A., Gattuso A., Ritorto D., Piscopo A., Poiana M. (2023). Effect of edible coating enriched with natural antioxidant extract and bergamot essential oil on the shelf life of strawberries. Foods.

[75] Pirozzi A., Del-Grosso V., Ferrari G., Donsì F. (2020). Edible coatings containing oregano essential oil nanoemulsion for improving postharvest quality and shelf life of tomatoes. Foods.

